# O10.2. PSYCHOTIC EXPERIENCES ARE ASSOCIATED WITH HEALTH ANXIETY AND FUNCTIONAL SOMATIC SYMPTOMS IN PRE-ADOLESCENCE

**DOI:** 10.1093/schbul/sby015.254

**Published:** 2018-04-01

**Authors:** Martin K Rimvall, Cecilia Pihl Jespersen, Lars Clemmensen, Anne Mette Skovgaard, Frank Verhulst, Jim van Os, Charlotte Ulrikka Rask, Pia Jeppesen

**Affiliations:** 1 Child and Adolescent Mental Health Center, Mental Health Services, The Capital Region of Denmark; 2 Research Clinic for Functional Disorders and Psychosomatics, Aarhus University Hospital; 3 University of Copenhagen; 4 Maastricht University Medical Centre

## Abstract

**Background:**

Psychotic experiences (PE) in children and adolescents include hallucinations, delusions and thought-disturbances in the absence of psychotic disorders. Psychosis can be viewed on a continuum ranging from subclinical PE throughout the life span, to clinical psychosis syndromes. Psychosis and PE often co-occur with anxiety and depression, and several studies point towards an affective pathway to psychosis.

Health anxiety (HA) is a relatively new concept in child and adolescent psychiatry, characterized by obsessive rumination, with thoughts about suffering from a disease and misinterpretation of benign bodily sensations and changes. HA at age 11–12 years are associated with emotional disorders and functional somatic symptoms (FSS). In adolescence extensive physical changes occur, and it has been suggested that increased bodily awareness in some cases is accompanied aberrantly by anxiety regarding somatic sensations and somatic health.

We hypothesized that PE would be associated with HA and FSS, and that the associations would remain significant after adjustment for general psychopathology, suggesting a particularly strong specific link between these specific psychopathologies over and above the general multidimensionality of psychopathology.

**Methods:**

The study population consists of 1572 children from the general population who participated in the 11–12 year follow-up of the Copenhagen Child Cohort 2000 (CCC2000). PE were assessed face-to-face by the Kiddie Schedule for Affective Disorders and Schizophrenia present and life-time version, and were rated dichotomously as either present (likely or definitely) or not present. HA was self-reported using the Childhood Illness Attitude Scale and FSS were self-reported using the Children’s Somatization Inventory, Child Report Form, revised. HA and FSS were scored dichotomously into high (high 10%) and low (bottom 90%) scores. The associations between PE and HA + FSS were adjusted for i) general psychopathology, rated by parents, using the Strengths and Difficulties Questionnaire total score, ii) chronic physical conditions assessed by parent report, iii) onset of puberty onset defined by Tanner-stage I vs II-IV and iv) sex.

**Results:**

PE were associated with HA (OR 2.91 (CI95% 1.86–4.57)) and FSS (OR 4.61 (CI95% 3.08–6.89)) in univariate analyses. In a mutually adjusted multivariate model which was further adjusted for general psychopathology, puberty, chronic physical conditions and sex, the associations still held significance for both HA (OR 1.73 (CI95% 1.03–2.90)) and FSS (OR 3.39 (CI95% 2.15–5.35)).

**Discussion:**

Our study is, to our knowledge, the first to estimate the role of HA and FSS with regard to PE. Our hypothesis, that PE are associated with HA and FSS in pre-adolescence, was confirmed. The statistical effects were reduced, but remained significant after mutual adjustment and adjustment for general psychopathology. This shows that part of the association is confounded by a general load of psychopathology, but also indicates that HA and FSS contribute to PE over and above general psychopathology. Our study warrants further longitudinal studies, exploring if HA and FSS might constitute a specific pathway in psychosis development.

